# The Immediate Analgesic Effect of Acupuncture for Pain: A Systematic Review and Meta-Analysis

**DOI:** 10.1155/2017/3837194

**Published:** 2017-10-25

**Authors:** Anfeng Xiang, Ke Cheng, Xueyong Shen, Ping Xu, Sheng Liu

**Affiliations:** Shanghai University of Traditional Chinese Medicine, Shanghai 200032, China

## Abstract

Although acupuncture is gaining popularity for the treatment of nonspecific pain, the immediate analgesic effect of acupuncture has never been reviewed. We conducted a systematic review and meta-analysis of randomized controlled trials (RCTs) on disease-related pain to critically evaluate the immediate effect of acupuncture for pain relief. The PubMed and Cochrane Central Register of Controlled Trials databases as well as three Chinese databases including the China National Knowledge Infrastructure (CNKI), Wanfang, and VIP platforms were searched through November 2016. The outcome was the extent of pain relief from baseline within 30 min of the first acupuncture treatment. We evaluated all RCTs comparing acupuncture with other interventions for disease-related pain. Real acupuncture showed statistically significantly greater pain relief effect compared to sham acupuncture (SMD, −0.56; 95% confidence interval [CI], −1.00 to −0.12; 9 RCTs) and analgesic injection (SMD, −1.33; 95% CI, −1.94 to −0.72; 3 RCTs). No serious adverse events were documented. Acupuncture was associated with a greater immediate pain relief effect compared to sham acupuncture and analgesic injections. Further RCTs with stricter design and methodologies are warranted to evaluate the immediate pain relief effect of acupuncture for more disease-related pain.

## 1. Introduction

Pain is a major health problem with serious social and economic consequences. The annual cost of pain management in the USA in 2010 was $560–635 billion, which was a conservative estimate because it excluded the cost of management of pain affecting institutionalized individuals [1]. Conventional medical treatments are only moderately effective, and they often cause adverse side effects. A majority of people suffering pain in the USA and Europe have reported inadequate pain control, and one-third worry about addiction to pain medications [2, 3]. Pain conditions appear to have a greater negative impact on the factors affecting the quality of life, such as work performance, sleep, and mood, compared with other health problems [4, 5]. Given the increasing life expectancy and the aging population, appropriate management of pain and reduction of disability are likely to assume greater importance.

Acupuncture, which is a mainstay in the healthcare practices of traditional Chinese medicine, is commonly used for the treatment of pain. There is substantial evidence for acupuncture being effective in the treatment of acute [6–8] and chronic pain [9]. To date, over 80 systematic reviews have been conducted to assess the role of acupuncture and related therapies in the relief of pain. However, the results of these systematic reviews are far from unanimous. The majority of the reviews reported positive results for pain relief in low back pain and osteoarthritis by acupuncture [10–12]. Two recent systematic reviews [13, 14] examined the efficacy of acupuncture in the relief of cancer-related pain, and both reported positive results. The systematic review and meta-analysis by Lu et al. suggested that acupuncture was useful in decreasing postoperative pain [15]. However, the efficacy of acupuncture as a treatment of pain in other pain conditions such as neuropathic pain [16] or fibromyalgia [17] remains inconclusive. Ernst et al. conducted a review of reviews [18] and concluded that acupuncture is not effective in reducing pain.

Various factors, such as acupuncture manipulation [19, 20], acupuncture sensation [21], acupoint prescription [22], pathological status [23], and types of pain [24], can affect the assessment of the therapeutic effect of acupuncture. The duration of acupuncture stimulation and acupuncture paradigm as well as the assessment of analgesic effect following acupuncture treatment in different clinical trials have been varied, and these time-dependent factors might be a crucial determinant in evaluating the analgesic effect of acupuncture. The effects of acupuncture can be classified as either the immediate effects (immediately after the end of the first treatment) or the cumulative effects of multiple acupuncture treatments [25, 26]. To date, most clinical trials and systematic reviews have focused on the cumulative analgesic effects. In fact, immediate analgesic effect could have clinical significance in determining the ultimate efficacy of acupuncture in pain management because of the following factors. (1) Psychological components such as conditioning and expectation may play important roles in acupuncture-induced analgesia. Patients who receive little benefit or no immediate analgesic effect following the first treatment might expect to be less likely to gain benefit from the subsequent treatment. (2) For many patients with acute postoperative pain and labor pain, the reported analgesic effect of acupuncture usually reflects the immediate effect. (3) The results of some fMRI studies have suggested that the immediate and cumulative acupuncture-induced analgesic effect elicit different temporal neural responses in a wide range of brain networks [27, 28], suggesting there is specific underlying mechanisms for the immediate analgesic effect of acupuncture.

To date, there is no published systematic review or meta-analysis of the immediate analgesic effect of acupuncture. Therefore, we conducted a systematic review and meta-analysis to evaluate the immediate effect of acupuncture for various disease-related pain in order to summarize the available evidence, evaluate the quality of that evidence, and offer suggestions for future research and treatment. This PRISMA-compliant (Table S1; see Table S1 in the Supplementary Material available online at https://doi.org/10.1155/2017/3837194) systematic review was conducted with the following aims: (1) to compare the immediate analgesic effect and safety of acupuncture in the treatment of disease-related pain with those of sham acupuncture and other active treatments; (2) to identify specific factors associated with positive results; and (3) to identify areas for future treatment and research.

## 2. Materials and Methods

### 2.1. Search Strategy

In the present study, we applied the review methods advocated by the updated Cochrane Handbook for Systematic Reviews of Interventions [29]. The protocol of this systematic review has been registered in PROSPERO (http://www.crd.york.ac.uk/PROSPERO/DisplayPDF.php? ID=CRD42016038154). We searched through the following databases to retrieve records from the earliest publications to those published till November 15, 2016: PubMed/MEDLINE, the Cochrane Central Register of Controlled Trials, and three Chinese databases including the China National Knowledge Infrastructure (CNKI), Wanfang, and VIP platforms. Acupuncture-related terms including acupuncture, electroacupuncture, and needle and pain-related terms including pain, ache, and analgesia were used as the key search terms in the English databases. The following key terms were used in the Chinese digital databases: zhenjiu, zhenci, dianzhen, zhen, and tong (which translate into acupuncture, needle-acupuncture, electroacupuncture, needle, and pain, resp.). Our search was restricted to trials published in English and Chinese. The reference lists of all of the retrieved trials and reviews were screened; relevant conference proceedings and abstracts as well as on-going and unpublished studies were also manually searched. Two reviewers independently evaluated each of the reports for eligibility. Disagreements were resolved by discussion.

### 2.2. Inclusion Criteria

Studies that met the following criteria were included in the present review: (1) randomized controlled clinical trials; (2) trials comparing acupuncture with sham acupuncture, no treatment, or effective western medications (e.g., anesthetics or analgesics); (3) studies including participants suffering from nonspecific pain; that is, there were no restrictions on the type, cause, or duration of pain; and (4) studies measuring self-reported pain relief using scales such as the visual analogue scale (VAS), numeric rating scale (NRS), or verbal rating scale (VRS). The outcome for this review was pain relief immediately following the first treatment (i.e., less than or equal to 30 min after the end of treatment) from the baseline level.

### 2.3. Exclusion Criteria

We exclude RCTs comparing different types of acupuncture among each other, or those using transcutaneous electrical nerve stimulation (TENS) as a treatment, or those of perioperative pain management, or those using pressure/palpation pain as the unique outcome.

### 2.4. Data Collection and Analysis

Two authors (AX and KC) independently extracted the study characteristics and outcome data from the included studies. Disagreements between the authors were resolved by discussion, and in case of continued disagreement, a third reviewer (SL) was consulted. Since the outcome for our review was the improvement in pain immediately after the end of the first treatment, in case of RCTs reporting the outcomes at multiple time points after treatment, we used the data at the time point closest to the end of the treatment. In cases where only the final and baseline scores were available, we calculated the mean change of the score by subtracting the mean final value from the mean baseline value and computed the change-from-baseline standard deviation using a correlation coefficient [43]. In cases where only the confidence intervals (CIs) were available, we computed the CIs for the mean values to calculate the standard deviations [44]. In cases where the values were only available in figures, we used a ruler to measure the value of the pain outcomes. We combined the results of groups in which the real acupuncture was adopted [36] to create a single pairwise comparison according to the Cochrane Handbook for Systematic Reviews of Interventions [43].

### 2.5. Assessment of Risk of Bias in the Included Studies

For each of the included studies, we assessed the risk of bias using the Cochrane Collaboration's risk of bias tool [45], which evaluates seven factors that might increase the risk of over- or underestimating an intervention effect.

In the assessment of the blinding of the participants and assessors, we assigned sham-controlled trials a judgment of “unclear” unless we were certain that the sham control was convincing enough in fully blinding the participants to the treatment being evaluated. We considered sham-controlled trials as having a low risk of bias for blinding if the RCT either (i) evaluated the credibility of the sham treatment and found it to be indistinguishable from true acupuncture or (ii) used a penetrating sham needle or a previously validated sham needle (e.g., the Streitberger needle [46]). Two of the authors (KC and AX) independently judged the risk of bias for each domain. Any disagreement was resolved by discussion.

### 2.6. Assessment of Adequacy of Acupuncture

Two acupuncturists (SL and XS) with a combined clinical experience of nearly 40 years in treating the pain syndrome with acupuncture, and who had previously worked on RCTs of acupuncture, assessed the adequacy of the acupuncture administered in the trials. Four aspects of each acupuncture intervention were assessed for adequacy: the choice of acupuncture points, needling technique, duration of treatment, and experience of the acupuncturist [47]. The total number of sessions and treatment frequency were not taken into account in the assessment since, according to the definition of the immediate effect described above, only the first treatment mattered in the present study. The likelihood of the sham intervention having physiological activity was also assessed by means of an open-ended question. The acupuncturist assessors were provided with only those parts of the publications that described the acupuncture and sham procedures so that their assessments could remain uninfluenced by the results of the trials. To test the success of blinding of the assessors to the study publication and results, we asked the assessors to guess the identity of each study being assessed. The acupuncturists assessed the adequacies independently and achieved consensus by discussion.

### 2.7. Data Synthesis and Statistical Analysis

We only pooled the data from the trials that used similar controls (e.g., sham acupuncture, no treatment, or drug injection treatment). For the pooled data, the summary test statistics were calculated with the RevMan software, version 5.1 [48], using the random effects model to account for the expected heterogeneity. We evaluated the heterogeneity using the *I*^2^ statistic [49], which indicates the proportion of variability across the trials not explained by chance alone [50]. The statistical heterogeneity was assessed using the *I*^2^ statistic; an *I*^2^ statistic value of 50% or more was considered as indicating substantial heterogeneity. All continuous data reported for all of the studies were represented in forest plots. We did not carry out meta-analysis when there were less than two studies in a comparison according to the definition of meta-analysis [50].

We analyzed the penetrating and nonpenetrating sham acupuncture-controlled trials separately; however, in cases where there were no large or significant differences in the pooled effect between these two subgroups of trials, we pooled the data of all such trials available.

Two of the authors (AX and KC) independently graded the overall quality of the evidence for each outcome using the Grading of Recommendations Assessment, Development and Evaluation (GRADE) classification [51].

### 2.8. Measures of Treatment Effect

The major outcomes of the review were the standardized mean differences (SMDs) in the pain outcomes between acupuncture and each of the control groups. We used the SMDs as the principal measure of effect size because although the RCTs had assessed the same outcomes, they had performed the measurements using different scales (e.g., VAS and NRS).

### 2.9. Subgroup Analysis

We performed the subgroup analysis of two clinical characteristics that might influence the immediate analgesic effect of acupuncture on pain: (1) the type of sham, penetrating or nonpenetrating; (2) the duration of pain, acute (≤3 months) or chronic (>3 months) [12]. We performed statistical tests for interaction only if each subgroup included more than one study. We calculated the *P* values, pooled estimates, and *I*^2^ values of each of the two relevant subgroups for the subgroup comparisons of both characteristics.

### 2.10. Sensitivity Analysis

Considering that clinical pain included in present study was induced by various diseases, we also conducted the sensitivity analysis using the leave-one-out approach. The study by Zhang et al. [42] was excluded for further meta-analysis.

## 3. Results

### 3.1. Search Results

**Figure 1:**
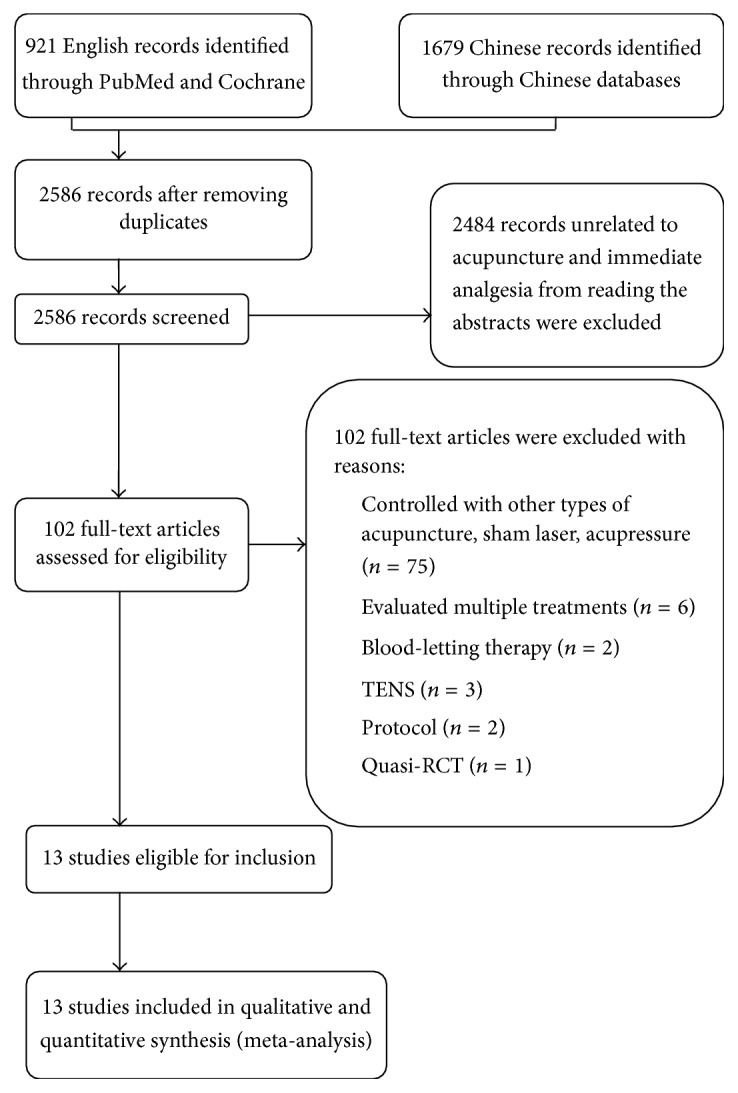
Flow diagram showing the number of studies included and excluded from the systematic review.

Figure 1 outlines the procedure of search and screening throughout the review. The initial electronic database search identified 2586 potential studies of interest. After screening these citations by their titles and abstracts, we considered 102 potentially eligible articles for inclusion and retrieved the corresponding full articles. Of the 102 studies, 89 were excluded because of the use of other types of acupuncture as controls, improper definition of the immediate effect, inaccurate protocols, or the quasi-random method of allocating patients to each group alternately, leaving 13 eligible RCTs [30–42]. Tables 1 and 2 describe the trial characteristics and the acupuncture and control interventions.

### 3.2. Characteristics of the Included Studies

We finally evaluated a total of 13 studies including a total of 1,077 participants with a mean age of 32 years (range, 20–78 years). For all of the eligible RCTs, the participants were required to have been diagnosed with disease-related pain for eligibility. Of the 13 RCTs, 4 investigated LBP [31, 32, 38, 40], and the remaining 9 investigated neck pain [35], neck and shoulder pain [37], carpal tunnel syndrome (CTS) [36], knee osteoarthritis [34], fibromyalgia [39], dysmenorrhea [33], sore throat [41], renal colic [30], and migraine [42]. The RCTs included in this review used either the VAS or NRS to measure pain outcomes. While 8 RCTs [30, 33, 36, 38–42] used acupoints based on the traditional Chinese medicine theory of meridians and collaterals, 4 [31, 32, 35, 37] used tender points near the most painful areas, and 1 [40] used points based on another acupuncture theory (i.e., the wrist-ankle acupuncture method). Electroacupuncture was administered in 5 RCTs [30, 33, 34, 36, 42] and manual acupuncture in 8 RCTs [31, 32, 35, 37–41]. Of the 9 RCTs that had used sham acupuncture as a control, 4 had used nonpenetrating sham [31, 36, 37, 40] and 5 had used penetrating sham [33, 34, 39, 41, 42]. Of the 3 RCTs that had used analgesic injection as a control, 2 [30, 38] had administered intramuscular analgesic injections and 1 [32] had administered a local anesthetic injection. One of the included RCTs [36] compared the effects of acupuncture at distal and local points and sham acupuncture; we, therefore, combined the results of the two real acupuncture groups to create a single pairwise comparison.

### 3.3. Acupuncture Adequacy

The acupoints and needling techniques were judged as being adequate in all of the included trials. All of the trials included in this review were judged to be adequate in terms of the treatment duration, except for those by Maeda et al. [36] and Yang et al. [41]. In the RCT by Maeda et al., only the fMRI scan time (5 min and 6 s) was known [36]. In the trial by Yang et al., the needle had been inserted and removed quickly [41]. Neither of the trials had reported the exact treatment durations, while the rest of the trials included in this review had. While the acupuncturists in 10 of the trials [32–38, 40–42] were judged as having adequate experience, we were unclear about the experience of the acupuncturists in the remaining 3 trials [30, 31, 39] owing to that fact that there is no description of the experience of acupuncturists in these studies. The assessors of acupuncture adequacy in this review were successfully blinded to all included publications and were unable to distinguish the origins of the results included.

### 3.4. Risk of Bias in the Included Studies

Of the 13 trials included in this review, 11 [30–35, 37–39, 41, 42] were assessed as having a low risk of bias upon sequence generation, while the risks of bias of the remaining 2 trials [34, 36] were assessed as being unclear (Table 1, Figures S1 and S2). The RCTs by Lu et al. and Maeda et al. claimed to have randomly assigned the participants but did not describe their methods in detail [34, 36]. Nearly half (6/13) of the included trials did not mention allocation concealment [30, 32, 34, 36, 37, 41] and were, therefore, assessed as having unclear risk of bias in this dimension. The remaining 7 trials were assessed as having a low risk of bias on allocation concealment. In one of the 9 sham-controlled trials [36], we were not certain whether the sham was distinguishable from true acupuncture by the participants because this trial used nonpenetrating sham acupuncture as a control, and the credibility of the sham had not been mentioned or evaluated in previous literature. The remaining 8 sham-controlled RCTs [31, 33, 34, 37, 39–42] had either used penetrating sham acupuncture or evaluated/mentioned nonpenetrating sham acupuncture in their study and were therefore assessed as having a low risk of bias on participant/assessor blinding. All of the included trials were regarded as having a low risk of incomplete outcome data (attrition bias) and selective reporting (reporting bias) because all of the patients had completed the first treatment session as well as the posttreatment assessment, and there had been no withdrawals.

### 3.5. Effects of Interventions

#### 3.5.1. Acupuncture versus Sham Acupuncture

**Figure 2:**
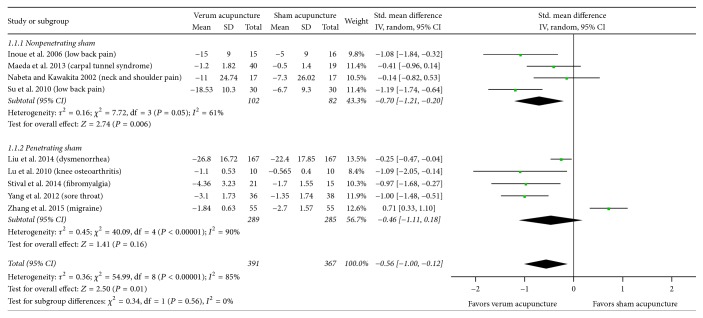
Acupuncture versus sham acupuncture: pain. 95% CI, confidence interval; Std., standardized.

Real acupuncture showed a greater immediate pain relief effect compared to sham acupuncture (SMD, −0.56; 95% CI, −1.00 to −0.12; 9 RCTs, Figure 2). There was a substantial heterogeneity of results in these trials (*I*^2^ = 85%). The results of the GRADE analysis indicated that the overall quality of evidence for this outcome was moderate as a consequence of uncertain risk of selection bias because of the nonavailability of detailed descriptions of sequence generation and allocation concealment (4 RCTs) and unclear risk of performance bias because of uncertain blinding (1 RCT).

#### 3.5.2. Acupuncture versus Analgesic Injection

**Figure 3:**
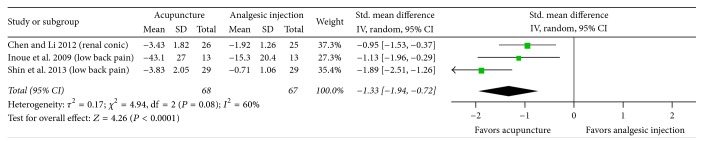
Acupuncture versus analgesic injection: pain. 95% CI, confidence interval; Std., standardized.

The results of the comparative efficacy studies revealed that acupuncture was associated with statistically significantly greater immediate pain relief compared to analgesic injection with nonsteroidal anti-inflammatory drugs (NSAIDs) or local anesthetic (SMD, −1.33; 95% CI, −1.94 to −0.72; 3 RCTs; Figure 3). There was a substantial heterogeneity of results in these trials (*I*^2^ = 60%). The results of the GRADE analysis indicated that the quality of evidence for this outcome was low as a consequence of a high risk of performance and detection bias because of inadequate data and the lack of blinding.

#### 3.5.3. Acupuncture versus No Treatment

Acupuncture was associated with statistically significantly greater immediate pain relief than no treatment (SMD, −1.63; 95% CI, 2.77 to −0.49; 1 RCT). The results of the GRADE analysis indicated that the quality of evidence for this outcome was low as a consequence of a high risk of performance and detection bias because of inadequate data and the lack of blinding.

### 3.6. Subgroup Analysis

**Figure 4:**
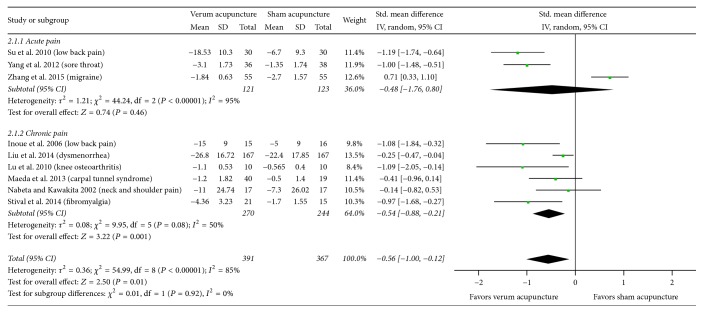
Subgroup analysis with the duration of pain (acute versus chronic) for sham-controlled trials. 95% CI, confidence interval; Std., standardized.

There was no statistically significant difference in the effect estimates between the two substrata for either of the clinical characteristics, that is, the type of sham (*P* = 0.56, Figure 2) and the duration of pain (*P* = 0.92, Figure 4). The results did show that true acupuncture was statistically significantly more effective than nonpenetrating sham acupuncture (SMD, −0.70; 95% CI, −1.21 to −0.20; 4 RCTs; *I*^2^ = 61%); however, the pooled result was not statistically significant when compared with that of penetrating sham acupuncture (SMD, −0.46; 95% CI, −1.11 to 0.18; 5 RCTs; *I*^2^ = 90%). For the duration of pain, the results showed that real acupuncture was statistically significantly more effective than sham acupuncture for chronic pain (SMD, −0.54; 95% CI, −0.88 to −0.21; 6 RCTs; *I*^2^ = 50%); however, the pooled result was not statistically significant for acute pain (SMD, −0.48; 95% CI, −1.76 to 0.80; 3 RCTs; *I*^2^ = 95%).

### 3.7. Sensitivity Analysis

**Figure 5:**
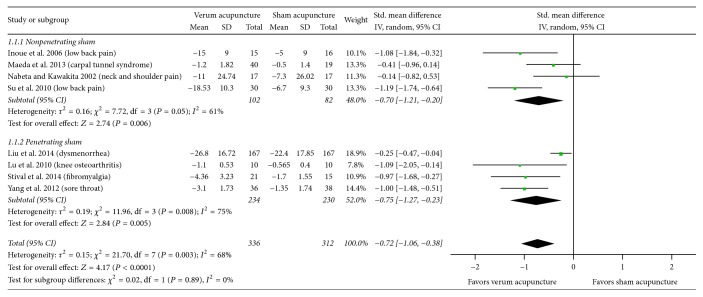
Subgroup analysis with the type of sham (nonpenetrating versus penetrating) for sham-controlled trials (excluding study by Zhang et al. [42]). 95% CI, confidence interval; Std., standardized.

**Figure 6:**
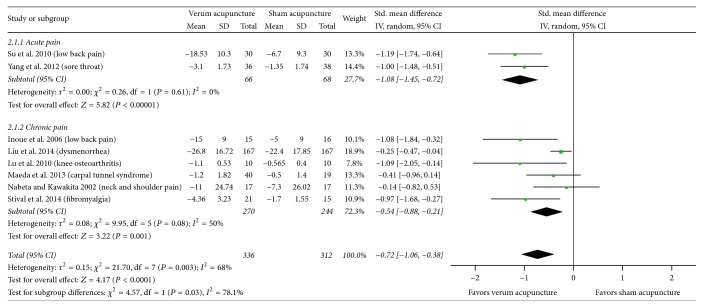
Subgroup analysis with the duration of pain (acute versus chronic) for sham-controlled trials (excluding study by Zhang et al. [42]). 95% CI, confidence interval; Std., standardized.

As shown in Table 3, heterogeneity of sham-controlled meta results decreased (*I*^2^ = 68%) when the study by Zhang et al. [42] was excluded. Real acupuncture consistently showed a greater immediate pain relief effect compared to sham acupuncture and drug injection by excluding Zhang et al.'s study (SMD, −0.72; 95% CI, −1.06 to −0.38; 8 RCTs, Figure 5) or each of the other included ones. After excluding the study by Zhang et al., subgroup meta-analysis showed that real acupuncture was better than penetrating sham in terms of the efficacy of pain relief (SMD, −0.75; 95% CI, −1.27 to −0.23; 4 RCTs, *I*^2^ = 75.8%, Figure 5), and acupuncture was more effective than the sham acupuncture in reducing acute pain (SMD, −1.08; 95% CI, −1.45 to −0.72; 2 RCTs, *I*^2^ = 0%, Figure 6). The heterogeneity of present study seems to be mainly from the Zhang et al.'s study.

### 3.8. Safety of Acupuncture

A total of 8 trials had included descriptions of adverse events associated with acupuncture [30, 32, 33, 35, 38, 40–42]. Seven of these 8 trials reported no adverse events following acupuncture treatment; only Liu et al. [33] reported a small hematoma in one of the patients in their acupuncture group and a small hematoma and needling pain experienced, respectively, in one patient in their penetrating sham acupuncture group. No serious adverse events were reported in any of the trials.

## 4. Discussion

This is the first systematic review and meta-analysis of RCTs on the immediate effects of acupuncture for the treatment of disease-related pain. We included a total of 13 studies in our review. The results showed statistically significant differences between the efficacy of real acupuncture and those of sham controls for all types of pain included in this review. The SMDs between real acupuncture and control sham acupuncture were lower than those between real acupuncture and a no-acupuncture control. In addition, acupuncture appeared to be more effective than analgesic injection (at intragluteal site with analgesic or local infiltration with anesthetic) in reducing pain. The meta-analytic effect sizes were not similar across pain conditions. There was no evidence of any significant harm caused by acupuncture in any of the RCTs. However, it should be stressed that this lack of evidence is based on the results of a few small trials with a high risk of bias. Therefore, a careful interpretation is warranted before arriving at a positive conclusion.

Compared with the assessment of the cumulative effects of acupuncture, the determination of the immediate effects could be relatively easy; that is, it is not necessary to consider the treatment endpoint or follow-up duration. Acupuncture also has a very low drop-out rate. For the systematic review and meta-analysis of the efficacy of acupuncture, various factors could affect the outcomes in the evaluation of the cumulative effects of acupuncture, including the total number of treatment sessions, treatment period, and variation in the end points, such as those of pain and function measurements at different times. Because of the exclusion or minimization of these variable factors, the evaluation of the immediate effect may closely reflect the actual analgesic effects of acupuncture stimulation.

Primary analgesic agents, such as morphine, can be used for the management of both acute and chronic pain. The peak effect of morphine is at around 20 min when administered intravenously and at 60 min when administered orally, while the duration of its effect is between 3 and 7 h [52, 53]. The results of our systematic review and meta-analysis indicate that acupuncture shows an immediate analgesic effect as the treatment of chronic pain. In general, the duration of onset of the effect of acupuncture is 15–30 min [15, 54]. The duration of the analgesic effect following a single session of acupuncture is about 3 days, although this duration is not consistent [55]. Therefore, the immediate effect of acupuncture may have clinical significance as an alternation for analgesic medication or as a reasonable method for pain treatment. Moreover, the success of acupuncture as a treatment of pain is often gauged by the number of clients retained in pain management or treatment facilities. The apparent benefits of the immediate analgesic effect of acupuncture may entice patients to receive long-term acupuncture treatment willingly or open to other forms of acupuncture options. Therefore, the immediate success of acupuncture treatment should not be overestimated. In present study, we did not find a greater immediate pain relief effect of acupuncture for acute pain compared with the sham acupuncture (*P* = 0.46). However, our sensitivity analysis showed that real acupuncture was more effective than the sham acupuncture in reducing acute pain immediately, if excluding the study by Zhang et al. [42]. This might be explained by some variable factors, such as the types of sham acupuncture, control procedures, and outcome measures. Further rigorous studies with standardized methodologies are required to test the efficacy of acupuncture for the treatment of acute pain.

The design of a control group is a continuing challenge for clinical trials of acupuncture. Many clinical trials were unable to detect statistically significant differences in the treatment efficacies between their acupuncture treatment and control groups in terms of any of the outcome measurements [56–58]; the authors of these trials concluded that acupuncture was no more effective than any sham interventions, for example, skin-touch sham (nonpenetrating) and skin-penetration sham in reducing pain. Based on the results of this systemic review and meta-analysis study, we found real acupuncture treatment has statistically significantly greater immediate pain relief than nonpenetrating sham acupuncture (SMD, −0.70; 95% CI, −1.21 to −0.20; 4 RCTs), but not these of penetrating sham acupuncture (SMD, −0.46; 95% CI, −1.11 to 0.18; 5 RCTs). Interestingly, when we excluded the study by Zhang et al. [42], we found real acupuncture was more effective than the sham acupuncture in relieving pain immediately after acupuncture treatment, which indicates some sham acupuncture treatment is not inactive.

Our systematic review and meta-analysis study focuses on the immediate analgesic effect of acupuncture. This raises some interesting questions. The first question is whether the immediately analgesic effect following the first acupuncture treatment can be used as a predictor for the success of subsequent or long-term acupuncture treatment. Most clinical trials focused on analgesic effects after multiple acupuncture treatment. Few studies assessed analgesic effects after both immediate posttreatment and multiple acupuncture treatments. Therefore, further studies must be performed to clarify this issue. The second question is whether the immediate acupuncture analgesic effect and cumulative analgesic effects following repeated acupuncture treatments share common mechanisms. Thus far, there is no clear documentation in regard to the underlying mechanisms of these two analgesic effects. Based on the available data published, needle insertion of the local acupuncture points triggers the release of adenosine and changes of fibroblast organization at the loose connective tissue layer [59–61]. The cumulative analgesic effects following repeated acupuncture treatments on the brain differ from the immediate analgesic effect after one acupuncture treatment. The immediate analgesic effect of acupuncture was a result of an extensive brain activation at selective pain-related regions [62]. However, the cumulative analgesic effects of acupuncture indicated bimodal habituation—a positive brain response appeared at the beginning of acupuncture stimulation, which then declined and became negative towards the final stages [28]. From neurohormonal prospective, a single acupuncture treatment can facilitate the release of opioid peptides [19]. Repeated administration of electroacupuncture leads to the development of opioid tolerance [63, 64]. Therefore, although acupuncture has both immediate and cumulative analgesic effects following repeated treatments, underlying mechanisms may be different.

Our systematic review and meta-analysis study has several limitations. Only English and Chinese literatures were reviewed in present study and potential data from studies published in other languages might exist and were ignored, which decreased the credibility of the results in present study to some extent. We included RCTs evaluating various types of pain, including chronic neck pain, LBP, and knee pain. In fact, accumulated work has shown that acupuncture is beneficial in treatment of various pain syndromes. The effects of acupuncture on nonspecific pain may share the similar underlying mechanisms. In traditional Chinese medicine, disease-related pain results from stagnation of energy “Qi” flow within meridians. Pain is treated locally or distally via acupuncture points further along the meridian, drawing energy away from the pain. Recently, the neurophysiology of acupuncture has been investigated extensively. Local anesthesia at the needle-insertion sites completely blocks the immediate analgesic effect of acupuncture, indicating that these effects are dependent on intact neural conduction. The immediate analgesic effect on various types of pain may be involved in the nociceptive pathway, including descending noradrenergic and serotonergic pathways [65]. In our meta-analysis, a high level of heterogeneity may be resulted from the baseline values, the acupuncture manipulation, acupuncture points selected, and the duration and frequency of treatment. Our review has a number of strengths. First, our search for relevant studies was extensive. Key Chinese databases were explored in addition to the English databases. Second, we assessed the differences in the immediate analgesic effect of acupuncture between real acupuncture and different types of controls. Third, the review only evaluated RCTs, which have study designs appropriate for the determination of the effects of intervention.

In conclusion, this review facilitates a better understanding of acupuncture stimulation and its immediate analgesic effect for disease-related pain. The results of our systematic review and meta-analysis suggest that evidence of the immediate analgesic effect of acupuncture is encouraging, but not convincing. Nevertheless, our review has yielded interesting and innovative findings and provided impetus to further investigations. Further rigorous, high-quality, randomized controlled trials comparing acupuncture with nontreatment and sham acupuncture without skin penetration are required to evaluate the immediate analgesic effect of acupuncture.

## Supplementary Material

Supplementary material included the risk of bias in present study. Risk of bias summary of included RCTs (Figures S1); The risk of bias assessment for each included study (Figures S2). As shown in Figures S1 and S2, all of the included trials were regarded as having a low risk of incomplete outcome data (attrition bias) and selective reporting (reporting bias).

## Figures and Tables

**Table 1:** Characteristics of randomized controlled trials.

Study, year	Country	Disease	*N* (M/F)	Mean age^*∗*^ (year)	Acupuncture (points; duration time)	Control	Time point after treatment for assessment	Pain assessment
Chen and Li [30] (2012)	China	Renal colic	51 (39/12)	39.72 (12.23)	EA (KI5, GB25, BL63, RN3, tender points; 30 minutes)	Intramuscular Fortanodyn injection	10 min	VAS

Inoue et al. [31] (2006)	Japan	Low back pain	31 (21/10)	69.03 (7.06)	MA (one most painful point; 20 seconds)	Nonpenetrating SA	Immediately	VAS

Inoue et al. [32] (2009)	Japan	Low back pain	26 (14/12)	72.20 (7.62)	MA ( two to five tender points; 20 seconds)	Local dibucaine injection	Immediately	VAS

Liu et al. [33] (2014)	China	Dysmenorrhea	501 (0/501)	22.40 (2.80)	EA (SP6; 30 minutes)	(1) EA at unrelated point (2) EA at SP	immediately	VAS

Lu et al. [34] (2010)	China	Knee OA	20 (unclear)	63.85 (5.95)	EA (GB34, SP9, SP10, ST34, ST36; 30 minutes)	Sham EA at SPs	Immediately	VAS

Mejuto Vázquez et al. [35] (2014)	Spain	Neck pain	17 (8/9)	24.53 (5.54)	DN (MTrPs; 25–30 seconds)	No treatment	10 min	NRS

Maeda et al. [36] (2013)	USA	CTS	59 (10/49)	49.1 (9.8)	EA (PC7, TW5 or SP6, LI4; more than 5 minutes)	Nonpenetrating SA	Immediately	VAS

Nabeta and Kawakita [37] (2002)	Japan	Neck and shoulder pain	34 (10/24)	32.5 (11.37)	MA (two to twelve tender points; 5 minutes)	Nonpenetrating SA	Immediately	VAS

Shin et al. [38] (2013)	Korea	Low back pain	58 (34/24)	38.31 (7.97)	Motion style acupuncture (DU16, LR2, LI11; 30 minutes)	Local diclofenac sodium injection	30 min	NRS

Stival et al. [39] (2014)	Brazil	Fibromyalgia	36 (5/31)	50.83 (9.51)	MA (PC6, HT7, SP6, LI4, LR2, ST36; 20 minutes)	Penetrating at SPs	Immediately	VAS

Su et al. [40] (2010)	China	Low back pain	60 (35/25)	39.6 (12.71)	MA (two ankle points; 30 minutes)	Nonpenetrating SA	Immediately	VAS

Yang et al. [41] (2012)	China	Sore throat	74 (37/37)	28.87 (13.78)	MA (LI4; removing the needle after eliciting the sensation)	Penetrating at SP	1 min	VAS

Zhang et al. [42] (2015)	China	Migraine	110 (52/58)	24.50 (3.03)	EA (GB20, SJ5, GB8, GB34; 30 minutes)	Penetrating at SPs	Immediately	NRS

*Notes.* ^*∗*^Mean (standard deviation); CTS: carpal tunnel syndrome; DN: dry needling; EA: electroacupuncture; F: female; M: male; MA: manual acupuncture; *N*: number; SA: sham acupuncture; SP: sham acupoint.

**Table 2:** Risk of bias summary.

Study, year	Sequence generation	Allocation concealment	Participants and assessor blinding	Treatment provider blinding	Incomplete outcome data addressed	Free of selective reporting	others
Chen and Li [30] (2012)	Low	Unclear^[b]^	High^[c]^	High	Low	Low	Low
Inoue et al. [31] (2006)	Low	Low	Low	High	Low	Low	Low
Inoue et al. [32] (2009)	Low	Unclear^[b]^	High^[c]^	High	Low	Low	Low
Liu et al. [33] (2014)	Low	Low	Low	High	Low	Low	Low
Lu et al. [34] (2010)	Unclear^[a]^	Unclear^[b]^	Low	High	Low	Low	Low
Mejuto-Vázquez et al. [35] (2014)	Low	Low	High^[d]^	High	Low	Low	Low
Maeda et al. [36] (2013)	Unclear^[a]^	Unclear^[b]^	Unclear^[e]^	High	Low	Low	High^[f]^
Nabeta and Kawakita [37] (2002)	Low	Unclear^[b]^	Low	High	Low	Low	Low
Shin et al. [38] (2013)	Low	Low	High^[c]^	High	Low	Low	Low
Stival et al. [39] (2014)	Low	Unclear^[b]^	Low	High	Low	Low	Low
Su et al. [40] (2010)	Low	Low	Low	High	Low	Low	Low
Yang et al. [41] (2012)	Low	Low	Low	High	Low	Low	Low
Zhang et al. [42] (2015)	Low	Low	Low	High	Low	Low	Low

^[a]^Lu et al. 2010 and Maeda et al. 2013 RCT claimed to have randomly assigned participants but did not describe the methods in detail; ^[b]^Chen and Li 2012, Inoue et al. 2009, Maeda et al. 2013, Lu et al. 2010, Nabeta and Kawakita 2002, and Stival et al. 2014 did not mention allocation concealment; ^[c]^Chen and Li 2012, Inoue et al. 2009, and Shin et al. 2013 compared acupuncture versus analgesia injection, and the participants, who were also the outcome assessors, could not be blinded; ^[d]^ Mejuto-Vázquez et al. 2014 compared acupuncture versus no treatment, and the participants, who were also the outcome assessors, could not be blinded; ^[e]^Maeda et al. 2013 RCT used nonpenetrating sham acupuncture as control but did not evaluate the credibility of the sham; ^[f]^for Maeda et al. 2013 RCT, the baseline was not comparable in the two groups.

**Table 3:** Sensitivity analysis of included studies.

Study, year	Statistics with study removed					
	Difference in means	Lower limit	Upper limit	*Z*-value	*P* value	*I* ^2^
*Acupuncture versus sham acupuncture*						
Inoue et al. 2006	−0.5	−0.97	−0.04	2.13	0.03	86%
Maeda et al. 2013	−0.58	−1.08	−0.09	2.31	0.02	87%
Nabeta and Kawakita 2002	−0.61	−1.10	−0.13	2.48	0.01	87%
Su et al. 2010	−0.47	−0.93	−0.02	2.06	0.04	84%
Liu et al. 2014	−0.62	−1.20	−0.04	2.10	0.04	87%
Lu et al. 2010	−0.51	−0.97	−0.05	2.17	0.03	87%
Stival et al. 2014	−0.51	−0.98	−0.04	2.14	0.03	86%
Yang et al. 2012	−0.50	−0.97	−0.03	2.09	0.04	85%
Zhang et al. 2015	−0.72	−1.06	−0.38	4.17	<0.0001	68%
*Acupuncture versus analgesic injection*						
Chen and Li 2012	−1.56	−2.30	−0.82	4.14	<0.0001	51%
Inoue et al. 2009	−1.41	−2.33	−0.49	3.00	0.003	79%
Shin et al. 2013	−1.00	−1.48	−0.53	4.12	<0.0001	0%
